# Myelomodulatory treatments augment the therapeutic benefit of oncolytic viroimmunotherapy in murine models of malignant peripheral nerve sheath tumors

**DOI:** 10.3389/fimmu.2024.1384623

**Published:** 2024-06-25

**Authors:** Siddhi N. Paudel, Brian J. Hutzen, Katherine E. Miller, Elizabeth A. R. Garfinkle, Chun-Yu Chen, Pin-Yi Wang, Andrea M. Glaspell, Mark A. Currier, Emily M. Ringwalt, Louis Boon, Elaine R. Mardis, Mitchell S. Cairo, Nancy Ratner, Rebecca D. Dodd, Kevin A. Cassady, Timothy P. Cripe

**Affiliations:** ^1^ Center for Childhood Cancer Research, The Abigail Wexner Research Institute at Nationwide Children’s Hospital, Columbus, OH, United States; ^2^ Institute for Genomic Medicine, The Abigail Wexner Research Institute at Nationwide Children’s Hospital, Columbus, OH, United States; ^3^ Department of Pediatrics, The Ohio State University Wexner College of Medicine, Columbus, OH, United States; ^4^ JJP Biologics, Warsaw, Poland; ^5^ Department of Pediatrics, Medicine, Pathology, Microbiology and Immunology, and Cell Biology, New York Medical College, Valhalla, NY, United States; ^6^ Cancer and Blood Diseases Institute, Cincinnati Children’s Hospital Medical Center, Cincinnati, OH, United States; ^7^ Department of Internal Medicine, Holden Comprehensive Cancer Center, University of Iowa, Iowa City, IA, United States; ^8^ Division of Pediatric Hematology/Oncology/BMT, Nationwide Children’s Hospital, Columbus, OH, United States

**Keywords:** malignant peripheral nerve sheath tumors, immunotherapy, tumor microenvironment, oncolytic virotherapy, macrophage targeting, trabectedin, pexidartinib, T-VEC

## Abstract

**Introduction:**

Malignant peripheral nerve sheath tumors (MPNST) pose a significant therapeutic challenge due to high recurrence rates after surgical resection and a largely ineffective response to traditional chemotherapy. An alternative treatment strategy is oncolytic viroimmunotherapy, which can elicit a durable and systemic antitumor immune response and is Food and Drug Administration (FDA)-approved for the treatment of melanoma. Unfortunately, only a subset of patients responds completely, underscoring the need to address barriers hindering viroimmunotherapy effectiveness.

**Methods:**

Here we investigated the therapeutic utility of targeting key components of the MPNST immunosuppressive microenvironment to enhance viroimmunotherapy’s antitumor efficacy in three murine models, one of which showed more immunogenic characteristics than the others.

**Results:**

Myelomodulatory therapy with pexidartinib, a small molecule inhibitor of CSF1R tyrosine kinase, and the oncolytic herpes simplex virus T-VEC exhibited the most significant increase in median survival time in the highly immunogenic model. Additionally, targeting myeloid cells with the myelomodulatory therapy trabectedin, a small molecule activator of caspase-8 dependent apoptosis, augmented the survival benefit of T-VEC in a less immunogenic MPNST model. However, tumor regressions or shrinkages were not observed. Depletion experiments confirmed that the enhanced survival benefit relied on a T cell response. Furthermore, flow cytometry analysis following combination viroimmunotherapy revealed decreased M2 macrophages and myeloid-derived suppressor cells and increased tumor-specific gp70+ CD8 T cells within the tumor microenvironment.

**Discussion:**

In summary, our findings provide compelling evidence for the potential to leverage viroimmunotherapy with myeloid cell targeting against MPNST and warrant further investigation.

## 1 Introduction

Malignant peripheral nerve sheath tumors (MPNST) are an aggressive form of soft tissue sarcoma of neuroectodermal origin that affects children and adults (1). The standard therapeutic approach for MPNST has been surgical resection with negative margins. However, the location of the tumor or size often limits this option, and chemotherapy is largely ineffective (2). A promising alternative cancer therapy being developed is oncolytic viroimmunotherapy, which selectively targets and eliminates tumor cells while triggering a robust antitumor immune response (3). In this study, we elucidate the potential of oncolytic herpes viroimmunotherapy as a therapeutic strategy for MPNST. The utilization of oncolytic viruses holds great promise in treating aggressive tumors like MPNST that have proven to be refractory to conventional therapies, owing to their capacity to induce durable and systemic immune responses by simultaneously targeting multiple hallmarks of carcinogenesis (4).

Preliminary studies have demonstrated that human MPNST cells exhibit high susceptibility to oncolytic viroimmunotherapy, which can be further enhanced by incorporating therapeutic transgenes such as *platelet factor 4 (PF4)*, an antiangiogenic factor, or *tissue inhibitor of matrix metalloproteinase-3 (TIMP3)*, a matrix metalloproteinase inhibitor (5–7). Additionally, encouraging results have been obtained by employing pharmacological interventions to enhance viral spread and counteract intrinsic interferon response-mediated antiviral resistance (8). However, despite these promising advances, attempts to integrate viroimmunotherapy with established treatments like erlotinib have failed to translate into therapeutic benefits (9). These results underscore an urgent need to address the barriers impeding the success of viroimmunotherapy in greater detail. Emerging evidence suggests that the tumor microenvironment plays a pivotal role in modulating the response to immunotherapies (10, 11). MPNST are characterized by an abundance of immunosuppressive myeloid cells as well as tumor-promoting cytokines/chemokines, and dysregulated expression of programmed death-ligand 1 (PD-L1) checkpoint ligands (12). These factors can impede the effectiveness of immune-based strategies (13–15). However, the influence of the MPNST tumor microenvironment on the therapeutic efficacy of oncolytic viroimmunotherapy remains unclear, as does the potential synergistic effect of targeted interventions aimed at the immunosuppressive microenvironment in combination with viroimmunotherapy. We seek to address this knowledge gap to establish a solid foundation for developing and optimizing oncolytic viroimmunotherapy as a potent therapeutic approach for MPNST.

**Figure 1:**
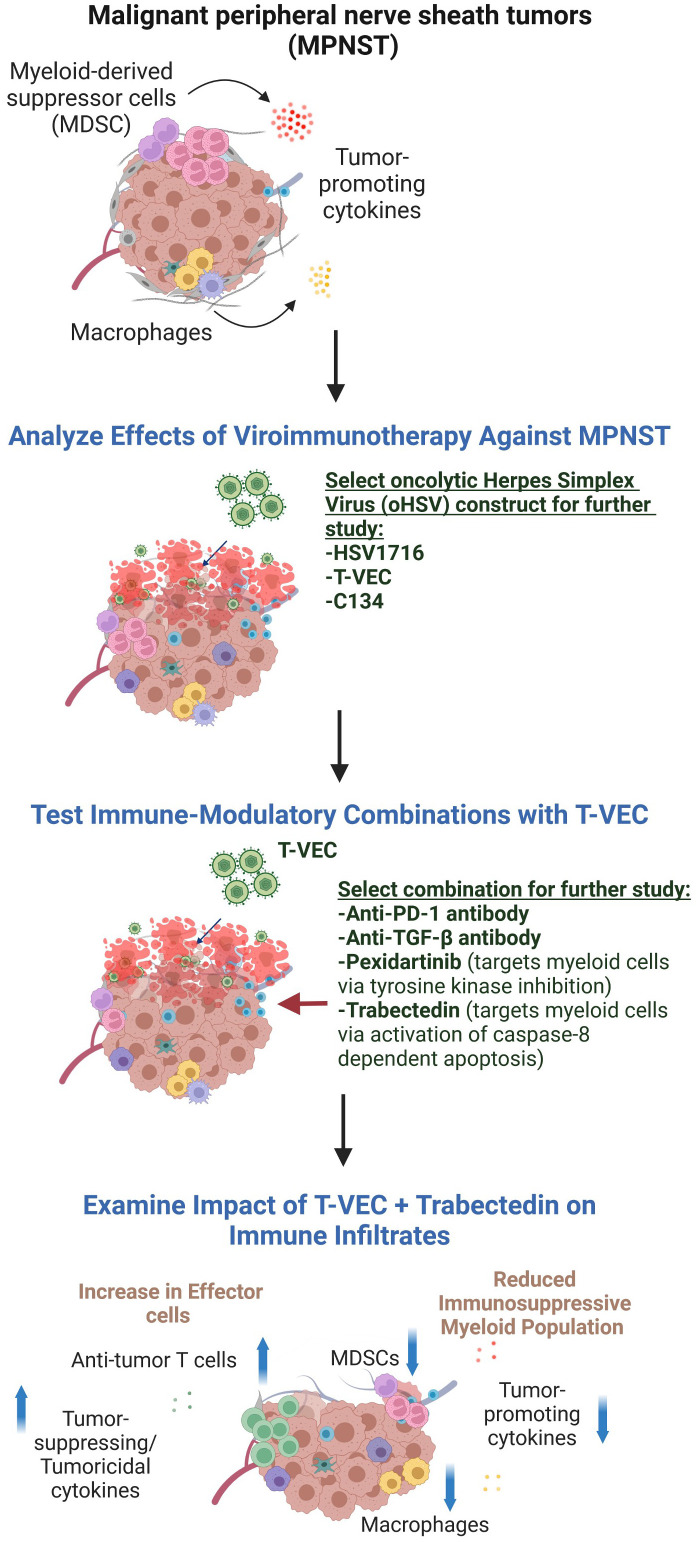
The experimental design used in this report. We first compared different oHSV constructs for their ability to kill MPNST cells and shrink MPNST syngeneic tumors. As none of the viruses was consistently superior to the others, we chose the FDA-approved virus, T-VEC, to further study in combination with drugs postulated to enable viroimmunotherapy. Because in other studies we found trabectedin to be toxic to C57Bl/6 animals, we pivoted to an MPNST model in the Balb/c background to test that combination. Figure created in BioRender.

We postulated that the therapeutic utility of viroimmunotherapy could be augmented through a rational combination with inhibitors of immunosuppression. To examine this hypothesis, we screened viroimmunotherapy combination regimens that target three key players of the immunosuppressive microenvironment: the transforming growth factor beta (TGF-β) signaling pathway, PD-1/PD-L1 immune checkpoint inhibitors, and myeloid cells (**Figure 1**). Thus, we employed numerous immunomodulatory therapies to alter the immune response to tumors, including mechanisms of specifically targeting myeloid cells – also termed myelomodulatory therapies. Previous studies on MPNST predominantly employed xenograft models, which have limitations in faithfully recapitulating the dynamics of immune responses to viroimmunotherapy. Consequently, our investigations focused exclusively on immunocompetent murine models of MPNST. Overall, our results indicate that myeloid cell-targeting therapies, also known as myelomodulatory therapies, can enhance the antitumor T cell response and improve the therapeutic benefits of oncolytic viroimmunotherapy in murine models of MPNST in the context of immunological heterogeneity.

## 2 Materials and methods

### 2.1 Cell lines

The murine MPNST cell lines 67C-4 and #5NPCIS obtained from the Ratner laboratory, were explanted from spontaneously arising tumors in Nf1:p53 mice mutated for NF1 and p53 (so-called NP-cis mice) (16, 17). Specifically, the 67C-4 cell line originated from female mice, while the #5NPCIS cell line was derived from male mice. These cell lines were cultured in Dulbecco’s Modified Eagle Medium (DMEM) supplemented with 10% heat-inactivated fetal bovine serum (FBS), penicillin (100 U/ml), and streptomycin (10 mg/ml). The murine MPNST cell line SN4-4, explanted from somatic CRISPR/Cas9 induced NF1/p53 null Balb/c murine tumor (18) and obtained from the Dodd laboratory, was cultured in DMEM supplemented with 10% FBS, sodium pyruvate (1 mM), penicillin (100 U/ml), and streptomycin (10 mg/ml). Prior to use, all cell lines were tested and confirmed to be negative for mycoplasma contamination by IDEXX Bioanalytics (Columbia, MO, USA).

### 2.2 Viruses

HSV1716 (SEPREHVIR™) was generously provided by Virttu Biologics (Glasgow, UK), presently under the ownership of Sorrento Therapeutics (San Diego, CA, USA). This virus strain was derived from a clinical isolate of herpes simplex virus (HSV) type 1 and features a deletion of a gene RL1 that encodes for the neurovirulence factor ICP34.5 (19). Talimogene laherparepvec (T-VEC) was procured from the research pharmacy at Nationwide Children’s Hospital (Imlygic™; Amgen, CA, USA). It is the only FDA-approved oncolytic virotherapy for intralesional injection in melanoma and is attenuated by deletion of RL1, similar to HSV1716. Moreover, T-VEC has a deletion of ICP47, which, during wild-type virus replication, reduces antigen presentation in infected human cells by impeding peptide loading into major histocompatibility complex (MHC) class I. Nevertheless, murine cells do not exhibit the ICP47-mediated inhibition of peptide loading into MHC class I due to the intrinsic species-specific nature of ICP47 (20). Additionally, T-VEC is engineered to express an immunostimulant cytokine hGM-CSF, although hGM-CSF is not functional in murine models (21). The C134 virus, originating from the Cassady Lab, was derived from an EGFP-expressing HSV mutant C101. This virus strain also harbors a deletion of ICP34.5 gene. Additionally, C134 also expresses a human cytomegalovirus IRS1 gene product that enables the virus to evade protein kinase R-mediated protein shutoff and sustain late viral protein synthesis (22).

### 2.3 Compounds and reagents

A8301 was obtained from Sigma-Aldrich (SML0788; St. Louis, MO, USA). It was reconstituted in Dimethyl sulphoxide (DMSO) to a final concentration of 5 mg/ml and then aliquoted and stored at -20°C. Pexidartinib, purchased from MedChemExpress (HY-16749; Monmouth Junction, NJ, USA), was reconstituted in DMSO to a final concentration of 125 mg/ml and stored in aliquots at -20°C. Trabectedin, also obtained from MedChemExpress (HY-50936; Monmouth Junction, NJ, USA), was reconstituted in DMSO to a final concentration of 4 mg/ml and stored in aliquots at -20°C. Recombinant murine interferon-gamma was purchased from PeproTech (315-05, Rocky Hill, NJ, USA).

### 2.4 Animals

C57BL/6 and BALB/c mice were procured from Envigo (Indianapolis, IN, USA). All animal studies were conducted in accordance with the guidelines set by the NIH Guide for the Care and Use of Laboratory Animals and with the approval of the Institutional Animal Care and Use Committee at Nationwide Children’s Hospital.

To generate murine tumors, subcutaneous injections of 5 x 10^6^ murine MPNST cells were administered into the flanks of 6-8-week-old C57BL/6 or BALB/c mice. Tumor size was measured regularly, either every other day or twice a week, using calipers. Tumor volumes were calculated using the formula: length x width^2^ x π/6. Upon reaching a size of approximately 150-300 mm^3^, the mice were randomly assigned to different treatment groups, ensuring comparable tumor burdens across the groups. Humane endpoints were defined as: a tumor volume of 2000 mm^3^, a tumor diameter of 2 cm, treatment-related ulceration > 1 cm in diameter, or 20% weight loss.

**Figure 2:**
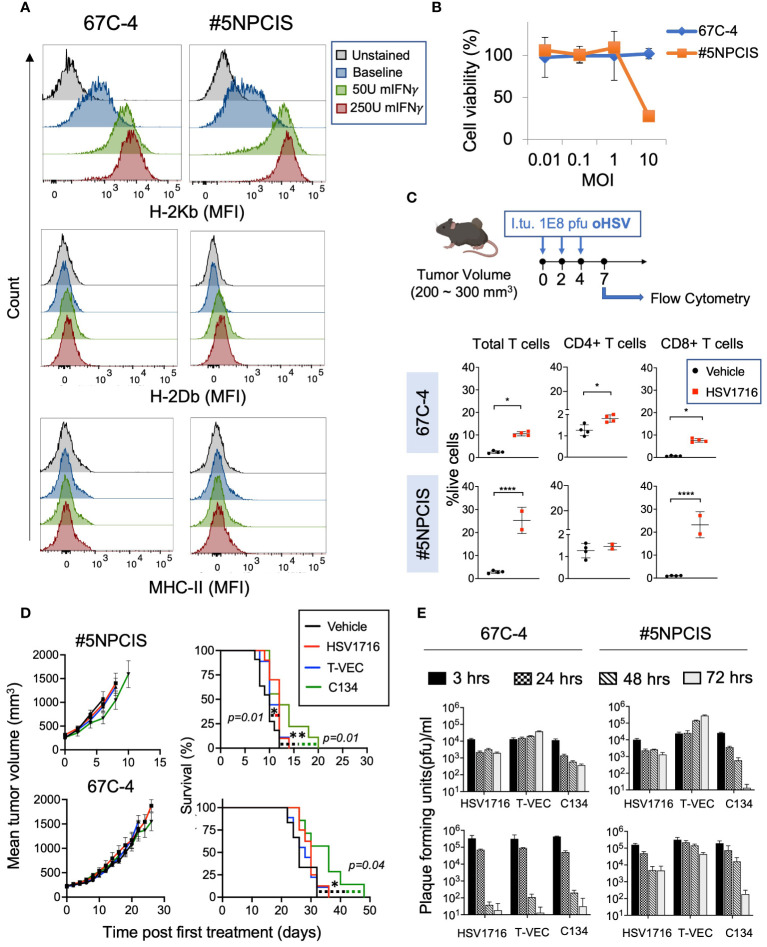
Comparison of three clinically relevant oncolytic herpes simplex viruses against MPNST. **(A)** Flow cytometry histograms of MHC Class I (H2-K^b^ and H2-D^b^) and MHC Class II in two murine MPNST cell lines, 67C-4 and #5NPCIS, at baseline and following exposure to murine Interferon-γ. Interferon-γ enhances the expression of H-2K^b^ in both cell lines. **(B)** MTS Viability Assay of MPNST cell lines following treatment with oncolytic herpes virus HSV1716 for 96 hours at varying multiplicity of infection (MOI). Samples were run in triplicate. Error bars represent SEM. **(C)** Dose scheduling of mice implanted subcutaneously with #5NPCIS or 67C-4 (top row). I.Tu., Intratumoral Injection; oHSV, oncolytic herpes simplex virus. Flow analysis of single-cell suspensions obtained from tumors treated with HSV1716 or vehicle control at day seven after the first treatment (bottom row). HSV1716 increases the infiltration of total T cells and CD8^+^ T cells by day 7 in both cell lines (*p≤0.05 and ****p≤0.0001). **(D)** Average tumor volume (left column) and Kaplan-Meier survival curve (right column) of #5NPCIS (top row) and 67C-4 (bottom row) treated with three clinically relevant oncolytic viruses at the dosing regimen same as panel. A modest but statistically significant impact was observed on overall survival with HSV1716 and C134 in the #5NPCIS model and C134 in the 67C-4 model. Error Bars represent SEM. Statistical significance was assessed using the log-rank test (n = 9-11 per group) (*p≤0.05 and **p≤0.01). **(E)** *In vitro* virus replication assays (top row) quantifying the amount of virus produced in 67C-4 and #5NPCIS cells after infection with HSV1716, C134 or T-VEC through plaque assays at an MOI of 0.5 plaque-forming units per cancer cell (n = 4 per group). T-VEC appears to have the highest permissivity. *In vivo* virus replication assays (bottom row) in mice bearing 67C-4 or #5NPCIS tumors treated with a single intratumoral 1 X 10^8^ pfu dose of virus (n = 4 per group). Error bars represent standard deviation. T-VEC had greater persistence than HSV1716 and C134 in the 67C-4 model.

For studies comparing three viruses, mice bearing 67C-4 or #5NPCIS tumor models were subjected to intratumoral fractionated injections of HSV1716, T-VEC, or C134. Each injection contained 1 x 10^8^ plaque-forming units (PFU) of the respective virus in 100 μl of phosphate-buffered saline (PBS). The control group received 100 μl of PBS alone via intratumoral injection. These injections were administered every other day for a total of three doses (**Figure 2**).

**Figure 3:**
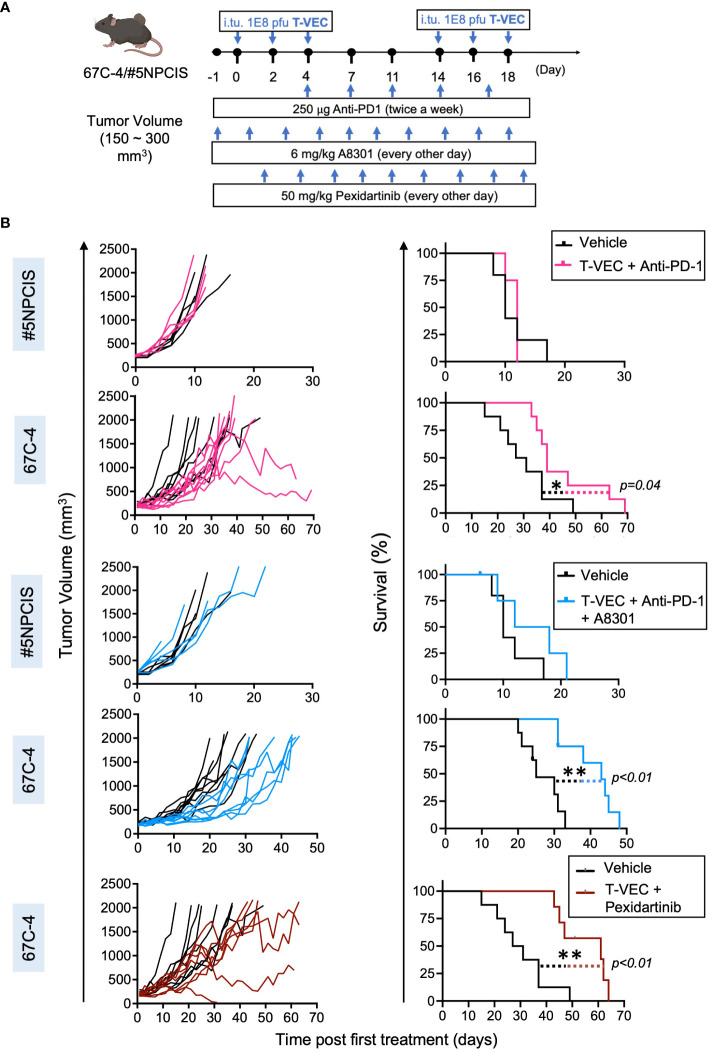
Screening of combination immunotherapy regimens that target three key players of the immunosuppressive microenvironment in MPNST to leverage virotherapy. Mice treated with the combination of T-VEC and pexidartinib had the greatest increase in median survival compared to other combinations. **(A)** Schematic of the treatment regimen of mice bearing subcutaneous 67C-4 or #5NPCIS tumors. I.Tu., Intratumoral Injection; oHSV, oncolytic herpes simplex virus. **(B)** Individual tumor volumes (left column) and Kaplan-Meier survival curve (right column) of #5NPCIS (top row of each combination) and 67C-4 (bottom row of each combination) following combination therapy. The bottommost row shows the combination of T-VEC and pexidartinib in the 67C-4 model. Combining T-VEC with either anti-PD-1 + A8301 or pexidartinib significantly prolongs survival in the 67C-4 tumor model. Among the tested combination regimens, no survival benefit was observed in the #5NPCIS tumor model. The statistical significance of survival data was assessed using the log-rank test (n = 4 to 5 for experimental groups including #5NPCIS mice; n = 7 to 8 for experimental groups including 67C-4 mice) (*p≤0.05 and **p≤0.01).

For the screening of combination immunotherapy regimens (**Figure 3**), mice were treated with intratumoral fractionated doses of T-VEC at a dose of 1 x 10^8^ PFU in 100 μl of PBS. The control group received 100 μl of PBS alone via intratumoral injection. The doses were administered every other day for a total of three doses, and the dosing regimen was repeated every two weeks.

For the study combining T-VEC and anti-PD-1 (RMP1-14), a subset of mice in each group received intraperitoneal injections of anti-PD-1 at a dose of 250 μg in 100 μl of PBS. The control group received 100 μl of PBS alone via intraperitoneal injection. The anti-PD-1 or PBS injections were administered twice a week. For the pexidartinib combination study, a subset of mice in each group received 100 μl oral gavage injections of 50 mg/kg pexidartinib, or PBS. These injections were administered every other day until the mice reached the endpoint. In the triple combination study of T-VEC, A8301, and anti-PD-1, all three drugs or the vehicle control were administered to a subset of mice in each group using the dosing regimen and route described above.

For studies testing the combination of T-VEC and trabectedin, mice received retro-orbital injections of trabectedin on Day 0 and 7, for a total of two doses and/or intratumoral fractionated doses of T-VEC at a dose of 1 x 10^8^ PFU in 100 μl of PBS. The control group received 100 μl of PBS alone via intratumoral injection. T-VEC was administered every other day for a total of three doses, and the dosing regimen was repeated every two weeks. Tumor volume was measured, and the mice were monitored until the tumors reached the endpoint volume of 2000 mm^3^, a diameter of 2 cm, or ulcers greater than 1 cm in diameter.

### 2.5 Depletion studies

For depletion studies, mice were treated intraperitoneally twice a week with 500 μg of anti-CD4 (GK1.5) and/or anti-CD8 (YTS169.4) antibodies, or an isotype control antibody (500 μg of anti-Phytophthora IgG AFRC MAC 51), in combination with the administration of T-VEC and trabectedin. A control group received 100 μl of PBS alone via intratumoral injection.

### 2.6 Flow cytometry analysis

Flow cytometry analysis was conducted as described previously (23). First, tumors were harvested and processed into single-cell suspensions through mechanical chopping, and incubation in 25 μg/ml liberase and 250 μg/ml DNAse I for 1 hour at 37°C. Then, tumor slurries were filtered using a 70 μm cell strainer, washed in fluorescence-activated cell sorting (FACS) buffer and treated with ammonium-chloride-potassium red blood cell lysis buffer (ThermoFisher A1049201). Subsequently, they were blocked with 5% mouse Fc blocking reagent (2.4G2, BD Biosciences, San Jose, CA, USA) in FACS buffer (1% FBS and 1 mM EDTA in PBS). Next, the cells were labeled with specific antibody staining panels on ice for 30 minutes to analyze innate and adaptive immune cells. The antibody panels used were as follows:

CD4-Fluorescein isothiocyanate (FITC) (GK1.5), CD25-phycoerythrin (PE) (7D4), CD69-allophycocyanin (APC) (H1.2F3), B220-PerCP (RA3-6B2), CD8a-PE-Cy7 (53-6.7), CD45.2-APC-Cy7, CD3ε-Violet 421 (145-2C11).

CD4-FITC, PD-L1-PE (10F.9G2), BALB/c gp70-TETRAMER APC, B220-PerCP, CD8a-PE-Cy7, CD45.2-APC-Cy7, CD3ε-Violet 421.

CD4-FITC, Lag3-PE (C9B7W), Tim3-APC (RMT3-23), B220-PerCP, CD8a-PE-Cy7, CD45.2-APC-Cy7, CD3ε-Violet 421.

CD4-FITC, CTLA4-PE (UC10-4B9), PD-1-APC (29F.1A12), B220-PerCP, CD8a-PE-Cy7, CD45.2-APC-Cy7, CD3ε-Violet 421.

CD4-FITC, CD49B-PE (DX5), CD44-APC (IM7), B220-PerCP, CD8a-PE-Cy7, CD62L-APC-Cy7 (MEL-14), CD3ε-Violet 421.

CD206-FITC (C068C2), Ly6C-PE (AL-21), MHC II-APC (M5/114.15.2), CD11c-PerCP/Cy5.5 (N418), F4/80-PE-Cy7 (BM8), Ly6G-APC-Cy7 (1A8), CD11b-Violet 421 (M1/70).

BALB/c Murine Leukemia Virus GP70 biotinylated monomer (H2-Ld-SPSYVYHQF) was obtained from the NIH tetramer core facility. This monomer was then tetramerized using streptavidin-APC to generate APC-conjugated GP70 as previously described (24). For Foxp3 intracellular staining, mononuclear cells were enriched by Percoll density gradient centrifugation (GE Healthcare Bio-Sciences, Pittsburgh, PA, USA). The cells were then washed with FACS buffer and blocked with 5% mouse Fc blocking reagent in FACS buffer. Subsequently, cells were stained with cell surface markers, including CD4-APC, CD8-PE-Cy7, CD25-PE (7D4), CD11b-PerCP, and CD3e-Violet 421. Intracellular staining for Foxp3-FITC (FJK-16s) was performed using a cell fixation and permeabilization kit (Invitrogen GAS001S100 and GAS002S100; ThermoFisher Scientific). After labeling, the cells were washed with FACS buffer and fixed in 1% paraformaldehyde. At least 100,000 events were collected and analyzed using BD FACS LSR II (BD Biosciences) and FlowJo Software, version 10.6.2 (Tree Star, Ashland, OR, USA).

For staining of two murine MPNST cell lines, the adherent cells were trypsinized and washed once with FACS buffer. They were then stained individually with H2-K^b^-PE (AF6-88.5.5.3), H2-D^b^-APC (KH95), and MHC-II-APC for 30 minutes on ice. The stained cells were washed with FACS buffer, fixed with 1% paraformaldehyde, and analyzed using FlowJo software.

Anti-Foxp3 and anti-H2-K^b^ antibodies were purchased from eBiosciences (San Diego, CA, USA), while the anti-Ly6C antibody was obtained from BD Biosciences. The remaining antibodies were purchased from BioLegend.

### 2.7 Cell viability assay

Three thousand cells were seeded in triplicate in each well of 96-well plates and incubated overnight at 37°C. The cells were then infected with oncolytic herpes simplex virus (oHSV) HSV1716 at different multiplicities of infection (MOI), including 0.01, 0.1, 1, and 10. Cell viability was assessed 96 hours post-infection using the CellTiter 96 Aqueous Non-Radioactive Cell Proliferation Assay (G5421; Promega, WI, USA) following the manufacturer’s instructions. The data were presented as the percentage of cell viability relative to the mock-infected controls. The error bars in the graph represent the standard deviation.

### 2.8 Virus replication assays

Virus Replication Assays were carried out as described previously (25). MPNST cells were cultured overnight in 12-well plates until they reached 80% confluency. The cells were then infected with HSV1716, C134, or T-VEC at an MOI of 0.5 for 3, 24, 48, and 72 hours. After the designated time points, the cells were scraped off the plates, collected into Eppendorf tubes, and subjected to three freeze-thaw cycles using dry ice. Next, the samples were centrifuged at 1500g for 15 minutes to remove the debris. The resulting supernatants were then serially diluted and titrated in triplicate on Vero cells, employing the standard plaque assay method.

In the *in vivo* experiments, mice with tumors ranging in size from 150-300 mm^3^ were administered a single dose of 1 x 10^8^ PFU of HSV1716, T-VEC, or C134. These mice were euthanized at 3, 24, 48, or 72 hours post-infection to collect the tumors. The harvested tumors were homogenized through mechanical disruption in 1 ml of DMEM. Subsequently, the homogenized samples were subjected to three freeze-thaw cycles on dry ice to disrupt the cells and release their contents. The samples were then centrifuged at 1500g for 10 minutes to separate the debris from the supernatant. The resulting supernatants were titrated on Vero cells in quadruplicate using plaque assays. The error bars in the analysis represent the standard deviation.

### 2.9 RNA sequencing and analysis

Tumor RNA was subjected to DNase treatment and ribodepletion prior to library construction using NEBNext Ultra II Directional RNA library prep kit for Illumina (New England BioLabs, Ipswich, MA, USA). Paired end 150 base pair reads were generated on the NovaSeq6000 to aim for a minimum of 80 million reads per sample; reads were aligned to the mouse genome reference sequence build mm10. Alignment was performed using a custom in-house pipeline and the splice-aware aligner STAR (26). The Salmon tool was used to quantify transcript abundance from the tumor RNA-seq reads generated (27). For retroviral transcript quantification, FASTQs were aligned and quantified, using salmon, to the gp70 sequence (GenBank: DQ359272.1).

To deconvolute and quantify immune cell populations from the RNA-seq data, we used the publicly available algorithm CIBERSORTx (28). The Salmon counts were referenced against the ImmuCC data set, a signature matrix file of 511 genes that accurately distinguish 25 mature mouse hematopoietic populations (29). Absolute immune cell type proportions, reported in absolute value, were plotted in GraphPad Prism Version 9.3.0 (GraphPad Software, Boston, MA, USA).

### 2.10 Whole exome sequencing

Exome sequencing libraries were prepared using the SureSelect non-human exomes preparation kit (Agilent). Target enrichment was performed using the SureSelectXT Mouse All Exon panel (Agilent #5190-4642) which captures all mouse exons (exon definition derived from Ensembl + RefSeq, designed against mm9 reference from UCSC). Libraries were generated using the NEBNext Ultra II FS Kit and paired end 151 base pair reads were sequenced on the NovaSeq6000 to aim for 100x coverage in normal tail sample comparators and 250x in tumor samples. Alignment was performed to the mouse reference genome build mm10 using a custom pipeline (30).

### 2.11 Statistical analysis

The significance of the difference in survival between treatment groups was assessed using log-rank Mantel-Cox tests. A survival event was defined as a tumor-related death. All nontumor-related deaths were demarked with tick marks on survival plots (censured data), but did not contribute to survival analysis. To determine if the differences observed in cell proportions through flow cytometry were statistically significant, a one-way ANOVA with Tukey-adjusted *post hoc* tests (>two groups) or a Student’s t-test (two groups) was utilized. All statistical analyses were conducted using GraphPad Prism Version 9.3.0 (GraphPad Software, Boston, MA, USA).

## 3 Results

### 3.1 Oncolytic herpes simplex viruses modulate the MPNST tumor immune microenvironment despite poor susceptibility to infection

First, we sought to determine if mouse models of MPNST respond to oncolytic viroimmunotherapy. We examined two murine MPNST cell lines, namely 67C-4 and #5NPCIS (**Table 1**). Since the loss of MHC Class I can compromise the efficacy of oncolytic viroimmunotherapy, we conducted flow cytometry analysis to determine the baseline expression of MHC Class I in these cell lines. We observed that both 67C-4 and #5NPCIS cell lines expressed the H2-K^b^ haplotype of MHC Class I at baseline and were capable of upregulating its expression upon exposure to interferon-gamma, a cytokine induced by most immune-based strategies as part of their anti-tumorigenic inflammatory response (**Figure 2A**) (31).

**Table 1:** MPNST mouse models’ characteristics and responses to viroimmunotherapy combination therapies.

MPNST model	Origin	Immunogenicity (relative)	Response to therapy combinations with viroimmunotherapy (T-VEC)
**#5NPCIS**	From a tumor that spontaneously arose in a male mouse with mutations in both the Nf1 and p53 genes (*C57BL/6 background*)	Moderate	•Anti-PD-1: No significant impact •Anti-PD-1 + TGF-βR1 inhibitor: No significant impact
**67C-4**	From a tumor that spontaneously arose in a female mouse with mutations in both Nf1 and p53 genes (*C57BL/6 background*)	High	•Anti-PD-1: Increased overall survival •Anti-PD-1 + TGF-βR1 inhibitor: Increased overall survival •Pexidartinib (myeloid-targeting): Increased overall survival
**SN4-4**	From a tumor that spontaneously arose in a mouse with CRISPR/Cas9 induced NF1/p53 mutations (*Balb/c background*)	Weak	•Trabectedin (myeloid-targeting): Increased overall survival

#### 3.1.1 Analysis of direct oHSV-mediated killing and oHSV-induced immune infiltration

Oncolytic viroimmunotherapy has two distinct modes of action: direct tumor cell lysis and the induction of an antitumor immune response (32). To assess the susceptibility of 67C-4 and #5NPCIS to HSV1716 virus-mediated lysis, we conducted cell viability assays (**Figure 2B**). While the #5NPCIS cell line exhibited susceptibility to HSV1716 killing at an MOI of 10, both 67C-4 and #5NPCIS displayed limited sensitivity to HSV1716 at an MOI of 1 and below (**Figure 2B**). This low susceptibility to oHSV is consistent with the limited susceptibility of murine models to most human oncolytic viruses (33).

Subsequently, as a preliminary assessment of the virus’s ability to induce an immune response, we evaluated intratumoral T cell infiltration following HSV1716 treatment in MPNST tumor-bearing mice. We implanted 67C-4 or #5NPCIS cells subcutaneously into gender-matched C57BL/6 mice and treated them with three intratumoral doses of HSV1716 or an equivalent volume of PBS when the tumors reached a volume of 200-300 mm^3^. Flow cytometry analysis of tumors on Day 9 after the initial treatment revealed a significant increase in T cells (CD4 and CD8) in the 67C-4 and #5NPCIS models (**Figure 2C**).

#### 3.1.2 Comparative efficacy of oHSV therapies in MPNST models

We next compared the therapeutic efficacy of three clinically tested oHSVs: HSV1716, T-VEC, and C134 (**Table 2**). We employed the same dosing regimen depicted in **Figure 2C** in 67C-4 and #5NPCIS murine models (**Figure 2D**). In our experiments, none of the oHSVs induced substantial tumor regressions for either tumor model. C134 exhibited a statistically significant increase in survival in both #5NPCIS and 67C-4 models, while HSV1716 demonstrated a significant increase in survival exclusively in the 67C-4 model. T-VEC had no statistically significant effect in these models. Overall, the impact of the tested oHSVs on tumor burden and survival was modest in both 67C-4 and #5NPCIS models (**Figure 2D**).

**Table 2:** Characteristics and outcomes of three clinically relevant oncolytic herpes simplex viruses against MPNST mouse models.

oHSV construct	Modification	Clinical application	Outcome against MPNST models
**HSV1716**	•ICP34.5 (*RL1)* deletion	Phase I trial displayed clinical safety in young patients (NCT02031965)	•Tumor burden: No significant impact •Survival: Increased in 67C-4 model •*In vitro* viral yield: Declined over time •*In vivo* persistence: Declined over time
**T-VEC**	•ICP34.5 (*RL1)* deletion •ICP47 deletion •Expresses the immunostimulant cytokine hGM-CSF	FDA-approved for melanoma	•Tumor burden: No significant impact •Survival: No significant impact •*In vitro* viral yield: Minimal loss in #5NPCIS model •*In vivo* persistence: Declined over time
**C134**	•ICP34.5 (*RL1)* deletion •Expresses human cytomegalovirus IRS1	Preclinical safety; Phase I trial ongoing for patients with glioma (NCT03657576)	•Tumor burden: No significant impact •Survival: Increased in #5NPCIS and 67C-4 models •*In vitro* viral yield: Declined over time •*In vivo* persistence: Declined over time

Additionally, we assessed the replication rates of these three oncolytic viruses in MPNST cells and tumors. In the *in vitro* experiments, HSV1716, C134, and T-VEC were administered to 67C-4 or #5NPCIS cell lines at a MOI of 0.5 PFU/cell. Virus production in these cell lines was quantified through plaque assays at designated time intervals post-infection (**Figure 2E**, top panel). T-VEC consistently exhibited an increasing trend in virus yield over the 72 hours, while both HSV1716 and C134 demonstrated a decline in virus production in both cell lines. These findings collectively establish T-VEC as the most permissive virus *in vitro*.

To validate these observations in our animal models, mice with subcutaneous 67C-4 or #5NPCIS tumors measuring 150-250 mm^3^ were treated with a single intratumoral dose of 1x10^8^ PFU of each virus. The mice were sacrificed at various time points, and the tumors were harvested for virus quantification using standard plaque assays (**Figure 2E**, bottom panel). In both 67C-4 and #5NPCIS tumor models, all three viruses gradually declined over time, with the decline being notably more pronounced in the 67C-4 model. Interestingly, T-VEC demonstrated enhanced persistence in #5NPCIS compared to HSV1716 or C134. However, this persistence did not impact the antitumor efficacy or animal survival (**Figure 2D**). In determining the primary candidate oHSV for our subsequent combination therapy experiments, we selected the FDA-approved T-VEC in order to investigate whether our combinatorial approaches could synergize with its superior persistence and achieve enhanced antitumor efficacy.

### 3.2 Myeloid-targeting via pexidartinib augments T-VEC efficacy in an immunogenic MPNST model

#### 3.2.1 Rationale for targeting immune checkpoint PD-L1

Immunosuppressive mechanisms within the tumor microenvironment limit the effectiveness of viroimmunotherapy (7). In MPNST, three primary immunosuppressive mechanisms have been identified: dysregulated cytokine/chemokine expression, infiltration of regulatory immune cells, and the presence of immune checkpoint molecules (12). To identify the most effective approach for leveraging viroimmunotherapy in MPNST, we screened three combination viroimmunotherapy regimens targeting these mechanisms in murine tumors.

MPNSTs express the immune checkpoint molecule PD-L1, which can impede the antitumor response of T cells (15, 34). In line with previous findings in colon and ovarian cancer models, we observed that oncolytic viroimmunotherapy could upregulate the expression of PD-L1 and enhance the proportion of PD-1^+^CD8 T cells in the tumor microenvironment (35, 36) (**Supplementary Figures 1A, B**). We then evaluated the efficacy of combining T-VEC with an anti-PD-1 antibody in MPNST tumor-bearing mice. We established the tumor models by subcutaneously implanting 67C-4 or #5NPCIS into gender-matched C57BL/6 mice, as described in **Figure 2**. Upon reaching a tumor size of 150-300 mm^3^, the mice were subjected to twice-weekly intra-peritoneal injections of 250 μg of anti-PD-1 antibody or an isotope control. Additionally, the mice also received three intratumoral doses of 1 X 10^8^ PFU of T-VEC or a corresponding volume of PBS every two weeks (**Figure 3A**). We observed a statistically significant increase in survival in the 67C-4 model but not in the #5NPCIS model for mice treated with T-VEC + anti-PD-1 (**Figure 3B**).

#### 3.2.2 Targeting TGF-β signaling pathway

MPNST cells exhibit an upregulation of immunosuppressive TGF-β ligands and a simultaneous decrease in TGF-β receptors, indicating a non-cell-autonomous effect of tumor-derived TGF-β on immune cells within the tumor microenvironment (37). This finding prompted us to investigate the therapeutic utility of combining TGF-β inhibition with viroimmunotherapy in MPNST, especially given prior reports of combination efficacy in other sarcoma models (25). Both 67C-4 and #5NPCIS cell lines exhibit detectable levels of TGF-β signaling, as demonstrated by immunoblot analysis showing phosphorylation of the downstream signal transducer SMAD2 (**Supplementary Figure 1C**). Addition of recombinant TGF-β results in a significant increase in SMAD2 phosphorylation, which can be reversed by the addition of 1 μM of TGF-β receptor 1 inhibitor A8301 (38). To evaluate TGF-β signaling inhibition *in vivo*, we administered 6 mg/kg A8301 or a vehicle control to mice bearing 67C-4 tumors every other day and collected tumor samples for immunoblot analysis on Day 5. Tumors treated with the vehicle control exhibited SMAD2 phosphorylation, whereas those treated with A8301 showed markedly reduced SMAD2 phosphorylation levels (**Supplementary Figure 1D**). Encouraged by these findings, we assessed the therapeutic potential of combining TGF-β receptor inhibition with T-VEC and anti-PD-1 treatments in mice with 67C-4 or #5NPCIS tumors. The mice received 6 mg/kg A8301 or a vehicle control every other day until reaching the experimental endpoint, along with a combination of T-VEC and anti-PD-1 (**Figure 3A**). In the 67C-4 model, the combination of A8301, anti-PD-1, and T-VEC significantly improved survival, prolonging the median survival time by 18 days compared to the vehicle control group. However, we did not observe any significant survival benefit in the #5NPCIS model (**Figure 3B**).

#### 3.2.3 Targeting myeloid cells with CSF1R inhibitor pexidartinib

Macrophage infiltration is a crucial immunosuppressive mechanism that can hinder the efficacy of immune-based therapies within the tumor microenvironment (39). In MPNST, there is a notable presence of immunosuppressive M2-like macrophages (13). The expression of activated colony-stimulating factor 1 receptor (CSF1R) has been observed in various MPNST cell lines, indicating a potential target for modulating macrophage activity (40). CSF1 is a vital cytokine involved in the regulation of monocyte/macrophage differentiation, proliferation, and survival, and its signaling is necessary for the accumulation of tumor-associated macrophages (41, 42). To address this barrier, we utilized pexidartinib, a small molecule inhibitor of CSF1R tyrosine kinase, in combination with viroimmunotherapy in the 67C-4 murine MPNST model, given the model’s immunogenic nature and previous sensitivity to our immune-modulating combination therapies (43). We treated mice bearing 67C-4 tumors with 50 mg/kg of pexidartinib or a vehicle control every other day until reaching the experimental endpoint. Additionally, these mice received three intratumoral doses of 1 X 10^8^ PFU of T-VEC every two weeks. Notably, the combination of T-VEC and pexidartinib demonstrated a significant increase in survival, with a median survival time extended by 32 days compared to the vehicle control group in the 67C-4 model. It is worth mentioning that pexidartinib, when administered as a monotherapy, did not exhibit any significant effect on the 67C-4 murine model (**Supplementary Figure 2**). Among the three combination regimens tested in the 67C-4 model, the combination of pexidartinib and T-VEC exhibited the greatest increase in median survival time, highlighting myelomodulatory treatment as an effective approach to leverage viroimmunotherapy in this model.

#### 3.2.4 Exploring underlying differences in response between 67C-4 and #5NPCIS models

**Figure 4:**
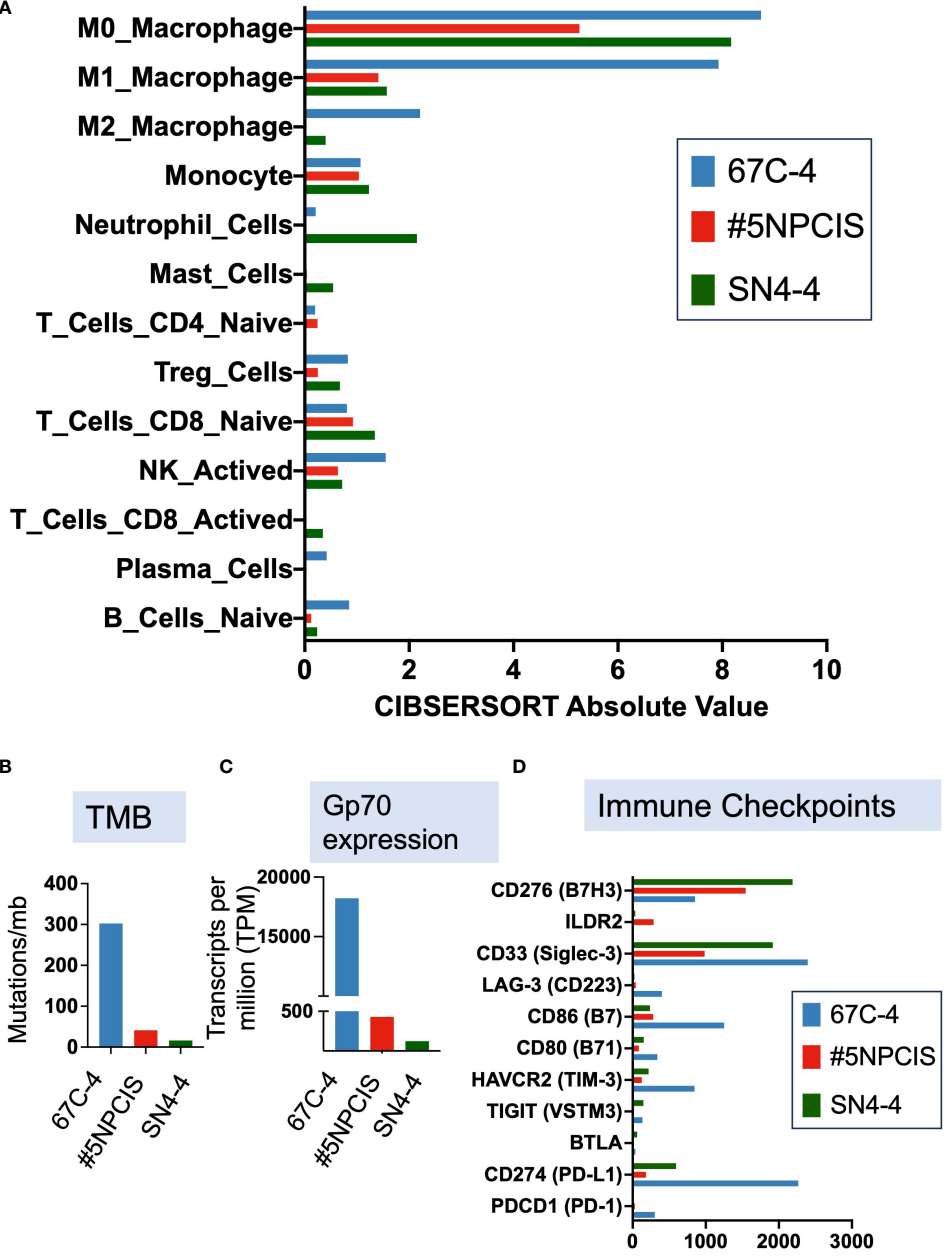
67C-4 has significant immune cell infiltration, higher tumor mutation burden, and greater expression of a known tumor-associated endogenous retrovirus antigen, envelope glycoprotein gp70, at baseline than #5NPCIS and SN4-4. **(A)** Deconvolution of RNA-sequencing data via CIBERSORTx to predict immune cell abundance in immunocompetent murine models. **(B)** Tumor Mutation Burden **(C)** gp70 expression **(D)** Immune Checkpoint expression (n=1-2 per tumor model).

To explore the factors contributing to the superior response of the 67C-4 model in contrast to the #5NPCIS model, we performed an RNA sequencing analysis on tumors extracted from mice bearing either 67C-4 or #5NPCIS tumors. Using CIBERSORTx analysis, we deconvoluted the RNA-sequencing data to estimate the abundance of 25 different types of mouse immunocytes. Our analysis revealed a substantial infiltration of immune cells in the murine tumors of the 67C-4 model, with myeloid cells dominating the population (**Figure 4A**). Additionally, the 67C-4 model exhibited a higher tumor mutation burden than the #5NPCIS model (**Figure 4B**). We also observed an elevated expression of the envelope glycoprotein gp70, a well-known tumor-associated endogenous retrovirus antigen, and immune checkpoints in the 67C-4 model compared to the #5NPCIS model (**Figures 4C, D**). Collectively, these findings strongly indicate that the 67C-4 model exhibits a greater degree of immunogenicity than the #5NPCIS model.

### 3.3 Myeloid-targeting via trabectedin synergizes with T-VEC through a robust T cell response

#### 3.3.1 Rationale for combining T-VEC with trabectedin

Given our encouraging data with pexidartinib, we sought to conduct further experiments to investigate if myeloid-targeting treatments could also enhance viroimmunotherapy in murine MPNST models with lower immunogenicity compared to 67C-4. However, the ability of pexidartinib to target a specific subset of suppressive myeloid cells, known as myeloid-derived suppressor cells (MDSC), is limited due to its demonstrated effect on altering chemokine expression by cancer-associated fibroblasts, which recruit more pro-tumor PMN-MDSC (44, 45). Based on recent single-cell RNA sequencing results showing activated tumor-associated fibroblasts in genetically engineered murine MPNST models, we chose to use the myelomodulatory drug trabectedin as a suitable alternative to treat the aggressive tumor (46). Trabectedin is FDA-approved for advanced liposarcoma and leiomyosarcoma and can deplete both macrophages and MDSC through TNF-related apoptosis-inducing ligand (TRAIL)-mediated apoptosis (47–49).

#### 3.3.2 Previous observations and selection of model

**Figure 5:**
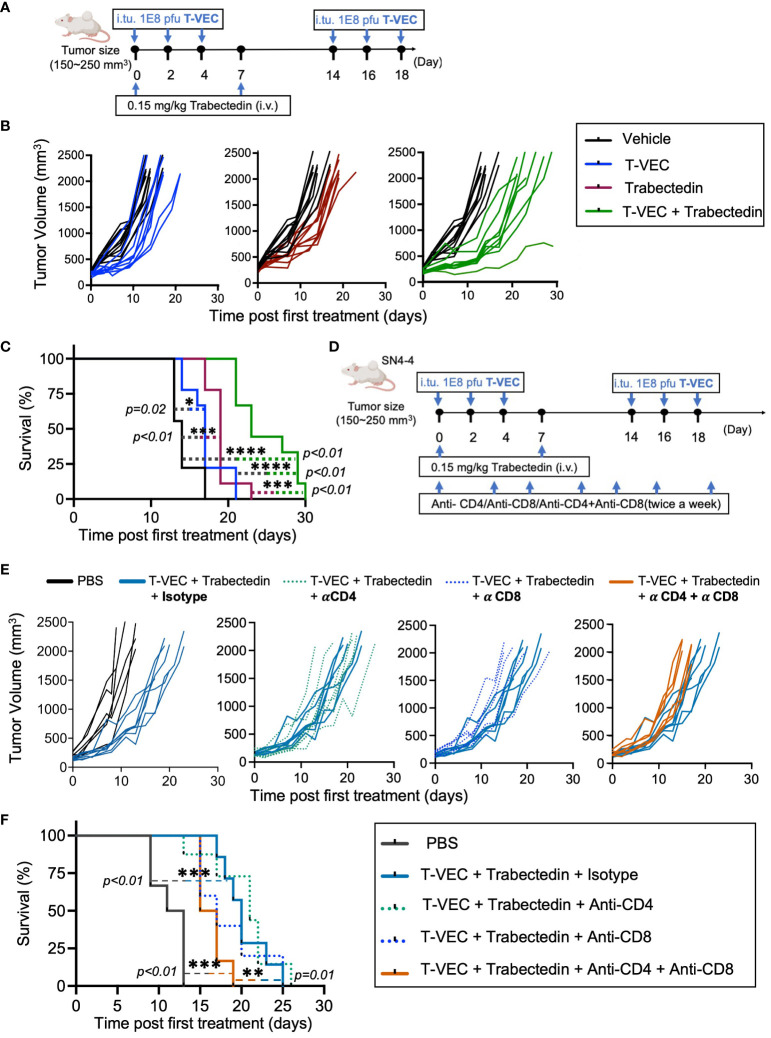
Trabectedin augments T-VEC virotherapy in a weakly immunogenic murine model of MPNST through a T cell response contributed by both CD4 and CD8 T cells. **(A)** Dose scheduling of mice implanted subcutaneously with SN4-4 tumors and treated with T-VEC, Trabectedin, or a combination of T-VEC and Trabectedin. I.Tu., Intratumoral Injection; oHSV, oncolytic herpes simplex virus.; i.v., Intravenous Injection **(B)** Individual tumor volumes of mice in each treatment group plotted against tumor volumes of control mice (black lines) (n=9 per group). **(C)** Kaplan Meier survival curves demonstrating prolonged survival in the combination group over monotherapies with T-VEC or trabectedin (n=9 per group) (*p≤0.05, ***p≤0.001 and ****p≤0.0001). **(D)** Schematic of the treatment regimen of mice bearing SN4-4 tumors undergoing combination therapy and administered with CD4 or CD8 T cell depleting or isotype control antibodies intraperitoneally twice a week (500 ug antibody per injection). **(E)** Individual tumor volumes of mice in each treatment group plotted against the tumor volumes of control mice undergoing combination therapy and treatment with isotype control antibodies (n = 7-10 per group). **(F)** Kaplan Meier survival curve demonstrating a decline in survival following treatment with CD4 and CD8 T cell depleting antibodies. The statistical significance of survival data was assessed using the log-rank test (n = 7-10 per group) (**p≤0.01 and ***p≤0.001).

Our previous observations revealed that combining oncolytic herpes simplex virus with trabectedin exhibited antitumor synergy in both xenograft (49) and immunocompetent models; however, it also led to hepatotoxicity in the C57BL/6 but not the BALB/c background (50). Consequently, we assessed the therapeutic potential of myeloid cell targeting with trabectedin in the weakly immunogenic SN4-4/BALB/c model of MPNST (**Figure 5**). SN4-4 cells were originally derived from MPNST tumors induced by injecting adenovirus containing Cas9 and guide RNA for Nf1 and p53 into the sciatic nerves, resulting in NF1/p53-null MPNST (**Table 1**) (18). For our experiments, we subcutaneously implanted SN4-4 cells into female BALB/c mice and initiated treatments when the tumor size reached 150-300 mm^3^.

#### 3.3.3 Efficacy of T-VEC, trabectedin, and combination therapy

Mice bearing SN4-4 tumors were given PBS or three intratumoral doses of T-VEC at a concentration of 1x10^8^ PFU on alternate days every two weeks (**Figure 5A**). Furthermore, subsets of mice from both groups were given two intravenous doses of trabectedin on days 0 and 7. Compared to the vehicle-treated group (**Figures 5B, C**, black line), the mice treated with T-VEC (**Figures 5B, C**, blue line) or trabectedin (**Figures 5B, C**, maroon line) showed delayed tumor growth and improved survival. There was a significant difference in survival benefit between these monotherapies and the vehicle-treated group. However, we did not observe a statistically significant difference between the effectiveness of T-VEC and trabectedin monotherapies. Encouragingly, the combination of T-VEC and trabectedin demonstrated enhanced survival benefit and antitumor efficacy compared to both monotherapies (**Figures 5B, C**, green line).

#### 3.3.4 Depletion of T cells abolishes combinatorial treatment benefit

To investigate whether the enhanced therapeutic response observed in the combination therapy was mediated by immune cells, we used monoclonal antibodies to selectively deplete CD4^+^ and/or CD8^+^ T cells in mice bearing SN4-4 tumors and undergoing combination therapy with T-VEC and trabectedin (**Figure 5D**). Notably, we observed a significant decline in survival benefit in mice depleted of CD4^+^ and CD8^+^ T cells, suggesting that both populations contribute to the enhanced efficacy of combination therapy (**Figures 5E, F**).

### 3.4 T-VEC and trabectedin combination therapy modulates the MPNST microenvironment to a pro-inflammatory phenotype

**Figure 6:**
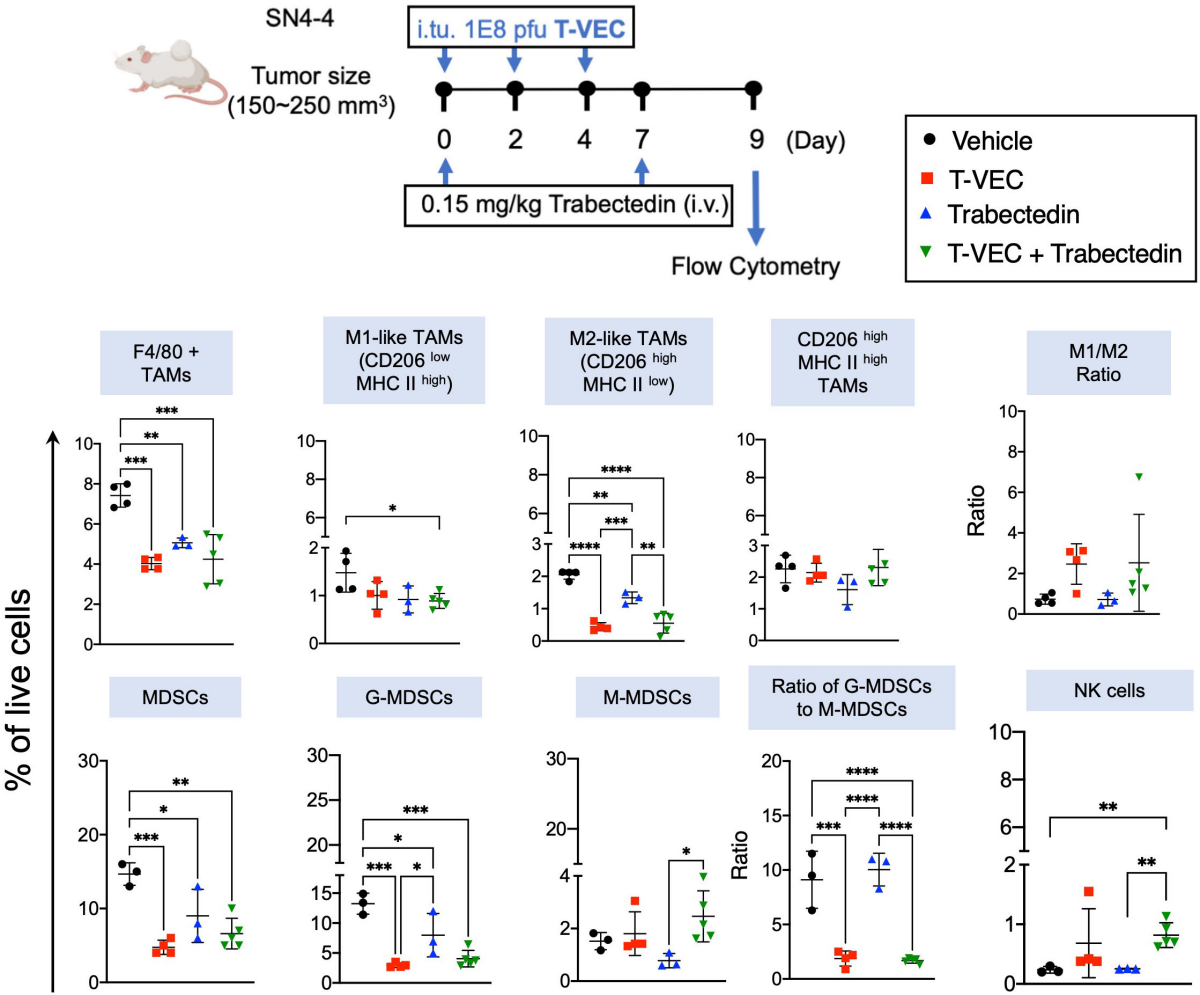
Combination of T-VEC and Trabectedin reduces the proportion of macrophages and myeloid-derived suppressor cells along with an increase in M1/M2 ratio similar to T-VEC. The proportion of NK cells increased significantly in the combination group over monotherapies. Schematic of the treatment regimen of BALB/c mice bearing subcutaneous SN4-4 tumors. The mice received a three intratumoral dose of T-VEC or an equivalent volume of PBS (Vehicle) on day 0, 2 and 4 (n=3-5 per group). Mice were sacrificed at Day 9 to harvest tumors for flow cytometry. Single-cell suspensions were obtained from the tumors, stained, and analyzed for macrophages, myeloid-derived suppressor cells and NK cells. G-MDSCs- Granulocytic-MDSCs; M-MDSCs-Monocytic-MDSCs. The statistical significance was assessed through a one-way ANOVA with Tukey-adjusted *post hoc* tests (*p<0.05, **p<0.01, ***p<0.001 and ****p<0.0001).

To better understand the influence of the combination of T-VEC and trabectedin on innate and adaptive immune cells, we evaluated intratumoral immune cell recruitment in mice bearing SN4-4 tumors following treatments as described in **Figure 5A**. On Day 9 post first treatment of the oncolytic virus, we harvested the tumors and processed them into single cell suspensions for flow cytometry (**Figure 6**).

#### 3.4.1 Analysis of myeloid cell markers

Initially, we stained a panel of myeloid cell markers (**Figure 6**). Our findings revealed that trabectedin significantly depleted the M2-like macrophage population (CD206^high^ MHCII ^low^). Interestingly, both T-VEC alone and the combination of T-VEC and trabectedin exhibited a reduction in M2-like macrophages, surpassing the decrease observed with trabectedin alone. Although the combination regimen induced a modest decrease in M1-like tumor-associated macrophages (CD206^low^ MHCII^high^), the effect was not as pronounced. Overall, all three treatment groups showed a significant decrease in the proportion of macrophages. However, it is essential to note that only the T-VEC-treated group or the combination of T-VEC and trabectedin demonstrated a trend towards an increased M1/M2 ratio compared to the vehicle-treated mice. This finding suggests a potential polarization of macrophages towards a pro-inflammatory antitumor phenotype in virotherapy groups, regardless of other concurrent therapies.

#### 3.4.2 Examination of MDSC markers

We then analyzed a panel of markers for MDSC (**Figure 6**). Remarkably, the proportion of MDSC was nearly two-fold higher than that of macrophages in this SN4-4 model. In addition, all three treatments significantly decreased the granulocytic subset of MDSC (G-MDSC), the predominant MDSC subset. However, the impact on monocytic MDSCs (M-MDSC) was not statistically significant, although trabectedin did show a trend toward a lower proportion of M-MDSC. Despite the decrease, the ratio of G-MDSC to M-MDSC remained unchanged in the trabectedin-treated group, suggesting the elimination of both immunosuppressive subpopulations. On the other hand, the virotherapy-treated group, regardless of concurrent trabectedin treatment, induced a significant decrease in the G-MDSC to M-MDSC ratio compared to the vehicle-treated or trabectedin-treated groups. This finding implies a specific impact of virotherapy on the balance between the two MDSC subsets, favoring a reduction in the immunosuppressive G-MDSC population.

#### 3.4.3 Evaluation of innate and adaptive effector immune cells

**Figure 7:**
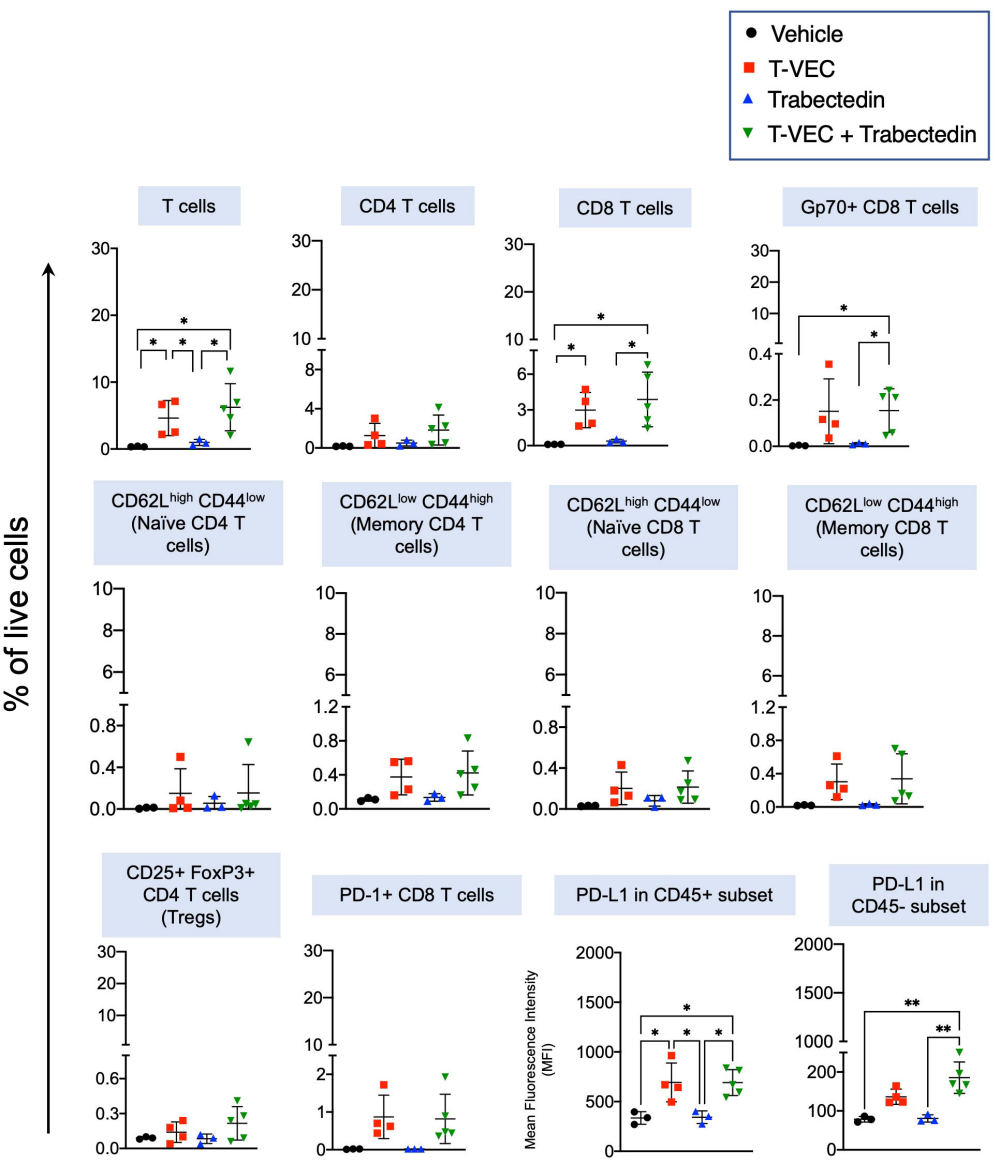
Combination of T-VEC and Trabectedin significantly increases the proportion of CD8 T cells specific to of a known tumor-associated endogenous retrovirus antigen, envelope glycoprotein gp70 compared to monotherapies with T-VEC or Trabectedin. CD8 T cells were significantly enhanced in the combination group, along with an increase in PD-L1 expression, while retaining the frequency of regulatory T cells similar to T-VEC treated groups. Single-cell populations were obtained from the same experiment described in **Figure 6** and stained for a panel of T cell markers (n=3-5 per group). The statistical analysis was performed through a one-way ANOVA with Tukey-adjusted *post hoc* tests (*p≤0.05 and **p≤0.01).

We then focused on assessing the innate and adaptive effector immune cells. A notable increase was observed in natural killer (NK) cells in the combination therapy group compared to both monotherapies and the control group (**Figure 6**). Additionally, the proportion of T cells significantly increased in the T-VEC-treated groups, regardless of concurrent trabectedin treatment (**Figure 7**). Trabectedin alone did not lead to an increase in the proportion of T cells. The proportion of CD4 T cells remained consistent across all treatment groups. On the other hand, CD8 T cells exhibited a significant increase in both T-VEC-treated and combination therapy groups (**Figure 7**).

Remarkably, only the combination of T-VEC and trabectedin significantly increased the proportion of T cells directed at the known tumor-associated endogenous retrovirus antigen, the envelope glycoprotein gp70, compared to the individual T-VEC or trabectedin treatments. We did not observe a significant change in the proportions of naive or memory CD4 and CD8 T cells, likely due to the weak immunogenicity of this model at baseline and low infiltration of T cells in the tumor microenvironment. The proportion of regulatory T cells remained low and unchanged across all treatment groups. There was a significant increase in the expression of PD-L1 in CD45^+^ immune cells within the virotherapy group, regardless of trabectedin treatment. A significant increase in PD-L1 expression was observed only in the CD45^-^ subset, which excludes immune cells. Overall, the data suggest that combining T-VEC and trabectedin enhanced the antitumor T cell response compared to the individual monotherapies.

## 4 Discussion

Our findings suggest that myeloid cell targeting can leverage the therapeutic utility of oncolytic viroimmunotherapy in murine models of MPNST. In two distinct murine models with varying degrees of immunogenicity, treatment with the myeloid-targeting agents pexidartinib or trabectedin significantly improved survival compared to T-VEC alone, which exhibited minimal or no survival benefit. The efficacy of the combination of T-VEC and trabectedin was dependent on both CD4 and CD8 T cells suggesting that trabectedin treatment augments T cell response in the tumor microenvironment. Flow cytometry analysis of the tumor microenvironment showed a significant increase in the proportion of CD8 T cells specific to the tumor-associated retrovirus antigen, envelope glycoprotein gp70, following combination viroimmunotherapy compared to monotherapies with T-VEC or trabectedin. Furthermore, the proportion of M2 macrophages and myeloid-derived suppressor cells decreased, while the M1/M2 ratio increased in the virus-treated and combination groups. These findings underscore the significant role of myeloid cells in mediating resistance to viroimmunotherapy in MPNST, limiting its antitumor efficacy.

We previously reported that myelolytic treatments with liposomal clodronate or trabectedin enhanced the antitumor efficacy of oncolytic herpes simplex virus rRp450 in xenograft models of Ewing sarcoma (49). rRp450 has impaired expression of viral ribonucleotide reductase, a key nucleotide metabolism enzyme that transforms ribonucleotides to deoxyribonucleotides, making the virus selective for cancer cells that contain a sufficient pool of nucleotides for replication, unlike normal cells. The Ewing sarcoma xenograft models tested were variably dependent on macrophages for tumor growth. The generalizability of the potential of myeloid cell targeting to augment viroimmunotherapy in two immunocompetent MPNST models with differing immune contexture presents a unique opportunity to expand the therapeutic utility of immune-based strategies in MPNST in the near future. For instance, T-VEC combinatorial treatments with anti-PD-1 and anti-PD-1 with TGF-β receptor 1 inhibition both displayed variable efficacy depending on the tumor model. This difference may be attributed to the immunogenicity of the models, given the differences we observed in infiltrating immune phenotypes – particularly macrophages – between tumor models. We acknowledge that other microenvironmental cells, such as stromal cells, may play a role in treatment efficacy differences; however, the presence of myeloid cells and the efficacy observed with myeloid-targeting therapies suggest that myeloid cells are important in tumor persistence despite PD-1 and TGF-β inhibition. Consistent with our results, a fibrosarcoma model poorly responsive to anti-PD-1 checkpoint inhibitor-based immunotherapy exhibited enhanced antitumor efficacy upon pretreatment with trabectedin (51). Although we did not combine anti-PD-1 along with trabectedin, both T-VEC and anti-PD-1 employ antitumor T cell response as their primary mechanism of tumor cell killing, suggesting that myeloid cell targeting has the potential to leverage the therapeutic utility of other immune-based strategies in MPNST. The combination of T-VEC and trabectedin did enhance PD-L1 expression in our murine models. Thus, it would be interesting to examine if incorporating PD-1 inhibition into our myeloid-targeting viroimmunotherapy combination regimen could surpass the therapeutic benefit observed with the combination of T-VEC and trabectedin. Along these lines, the triple combination of pexidartinib, oncolytic virus, and anti-PD-1 improved survival in murine models of colon cancer (52).

Contrary to our findings, the role of myeloid cells in therapy resistance may not be universal. This possibility is supported by evidence from syngeneic ovarian tumors, where combining chemotherapy with a CSF1R inhibitor (AZD7507) resulted in a decline in antitumor efficacy compared to chemotherapy alone (53). However, these observations may be attributed to the phenotypic variation of myeloid cells within the tumor microenvironment, which can also be polarized to promote an antitumor response. Specifically, in our models, the profile of macrophages broadly aligns with a tumor-promoting role.

Previous results suggested tumor-associated macrophages as potential orchestrators of tumor progression in MPNST (54). However, our findings indicate that myeloid cells, including macrophages and MDSC, also hinder the response to immune-based strategies. These results hold significant implications for the design of future combination immunotherapy regimens in the clinical setting. The therapies we examined in our study, including T-VEC, pexidartinib, and trabectedin, have already received individual FDA approval and are considered safe and well-tolerated for other medical indications. This fact presents a more direct and streamlined path for translation should further studies be initiated.

Even though we observed a reduced rate of tumor growth and enhanced survival, we failed to observe actual tumor shrinkages or cures. While we were limited in testing our approach of inhibiting MDSCs and macrophages using trabectedin in the more immunogenic model due to both the strain-specific toxicity of trabectedin in C57BL/6 mice and the lack of more immunogenic immunocompetent MPNST models in the BALB/c background, the combination of T-VEC and trabectedin likely expands the utility in these settings. However, our study’s ability to fully recapitulate the extent of viroimmunotherapy-induced oncolysis, subsequent immune activation against the tumor, and overall therapeutic efficacy has been constrained by the reduced permissiveness of murine tumors to oHSVs. Consequently, the therapeutic benefits observed from combination therapy in our weakly immunogenic model likely correspond to the advantage observed in the immune-desert microenvironment of MPNST tumors. It is plausible that myeloid-targeting combination therapy would greatly benefit patients with a higher degree of immunogenicity, in contrast to those with less immunogenic tumors.

The critical question from our study pertains to determining the optimal reduction in myeloid cell abundance to achieve clinical efficacy. To date, clinical trials investigating myeloid cell modulators have yielded limited success, even when combined with checkpoint inhibition. Hence, it is imperative to accurately identify the patient subtype(s) that our murine models mimic, as it holds significant value for translation. Recent findings regarding PRC2, a histone-modifying complex involved in transcriptional silencing, have revealed an immune cell-rich phenotype in tumors with PRC2 expression and vice versa. Exploring the influence of PRC2 status on myeloid-targeting combination viroimmunotherapy in MPNST might provide valuable insights (55). Moreover, stratifying more immunogenic tumors based on their baseline interferon response could harness the interplay between antiviral and antitumor immune responses, thereby enhancing the therapeutic efficacy of the combination therapy. In our less immunogenic models, we observed a higher abundance of MDSCs, specifically G-MDSCs, than macrophages. Interestingly, this MDSC dominance was effectively reduced through our myeloid-targeting treatment via trabectedin. Targeting G-MDSCs has proven to be particularly challenging due to the lack of identifiable and accessible targets for intervention. Therefore, trabectedin emerges as a potent modulator of myeloid cells in our model. The precise role of MDSCs and the significance of this shift in the macrophage-to-MDSC ratio or the G-MDSC-to-M-MDSC ratio, remains unknown and warrants further investigation.

### 4.1 Conclusion

In conclusion, our study provides compelling evidence for targeting myeloid cells to optimize viroimmunotherapy in MPNST. These findings expand the potential of immune-based strategies in this aggressive and treatment-refractory cancer and highlight new avenues for therapeutic enhancement. Further research in this area holds promise for advancing therapeutic approaches and ultimately improving outcomes for MPNST patients.

## Data availability statement

The datasets presented in this study can be found in online repositories. The link to view this data can be found below: https://www.ncbi.nlm.nih.gov/geo/query/acc.cgi?acc=GSE244829.

## Ethics statement

The animal study was approved by Institutional Animal Care and Use Committee at Nationwide Children’s Hospital. The study was conducted in accordance with the local legislation and institutional requirements.

## Author contributions

SP: Writing – review & editing, Conceptualization, Data curation, Formal analysis, Funding acquisition, Investigation, Methodology, Project administration, Resources, Software, Validation, Writing – original draft. BH: Conceptualization, Investigation, Methodology, Validation, Writing – review & editing, Supervision, Visualization. KM: Data curation, Software, Visualization, Writing – review & editing. EG: Visualization, Writing – review & editing. CC: Formal analysis, Investigation, Methodology, Validation, Writing – review & editing. PW: Formal analysis, Investigation, Methodology, Validation, Writing – review & editing. AG: Writing – review & editing, Investigation, Methodology, Validation. MC: Writing – review & editing. ER: Formal analysis, Visualization, Writing – review & editing. LB: Writing – review & editing, Investigation, Methodology, Validation. EM: Writing – review & editing, Data curation, Software. MC: Writing – review & editing. NR: Writing – review & editing. RD: Investigation, Methodology, Validation, Writing – review & editing. KC: Conceptualization, Funding acquisition, Project administration, Resources, Writing – review & editing. TC: Conceptualization, Funding acquisition, Project administration, Resources, Supervision, Writing – review & editing.
