# Corrigendum: Myelomodulatory treatments augment the therapeutic benefit of oncolytic viroimmunotherapy in murine models of malignant peripheral nerve sheath tumors

**DOI:** 10.3389/fimmu.2024.1456897

**Published:** 2024-07-09

**Authors:** Siddhi N. Paudel, Brian J. Hutzen, Katherine E. Miller, Elizabeth A. R. Garfinkle, Chun-Yu Chen, Pin-Yi Wang, Andrea M. Glaspell, Mark A. Currier, Emily M. Ringwalt, Louis Boon, Elaine R. Mardis, Mitchell S. Cairo, Nancy Ratner, Rebecca D. Dodd, Kevin A. Cassady, Timothy P. Cripe

**Affiliations:** ^1^ Center for Childhood Cancer Research, The Abigail Wexner Research Institute at Nationwide Children’s Hospital, Columbus, OH, United States; ^2^ Institute for Genomic Medicine, The Abigail Wexner Research Institute at Nationwide Children’s Hospital, Columbus, OH, United States; ^3^ Department of Pediatrics, The Ohio State University Wexner College of Medicine, Columbus, OH, United States; ^4^ JJP Biologics, Warsaw, Poland; ^5^ Department of Pediatrics, Medicine, Pathology, Microbiology and Immunology, and Cell Biology, New York Medical College, Valhalla, NY, United States; ^6^ Cancer and Blood Diseases Institute, Cincinnati Children’s Hospital Medical Center, Cincinnati, OH, United States; ^7^ Department of Internal Medicine, Holden Comprehensive Cancer Center, University of Iowa, Iowa City, IA, United States; ^8^ Division of Pediatric Hematology/Oncology/BMT, Nationwide Children’s Hospital, Columbus, OH, United States

**Keywords:** malignant peripheral nerve sheath tumors, immunotherapy, tumor microenvironment, oncolytic virotherapy, macrophage targeting, trabectedin, pexidartinib, T-VEC

In the published article, there was an error in the legend for Figure 1 as published. We mistaken referred to the tumor models as xenografts instead of syngeneic models in the sentence: “*We first compared different oHSV constructs for their ability to kill MPNST cells and shrink MPNST xenografts.*”

The corrected legend appears below.

*The experimental design used in this report. We first compared different oHSV constructs for their ability to kill MPNST cells and shrink MPNST syngeneic tumors. As none of the viruses was consistently superior to the others, we chose the FDA-approved virus, T-VEC, to further study in combination with drugs postulated to enable viroimmunotherapy. Because in other studies we found trabectedin to be toxic to C57Bl/6 animals, we pivoted to an MPNST model in the Balb/c background to test that combination.*

The authors apologize for this error and state that this does not change the scientific conclusions of the article in any way. The original article has been updated.

