# Supplementary material for: Myelomodulatory treatments augment the therapeutic benefit of oncolytic viroimmunotherapy in murine models of malignant peripheral nerve sheath tumors
Source: Front Immunol. 2024 Jun 25;15:1384623. doi: 10.3389/fimmu.2024.1384623 (PMC11263800; doi:10.3389/fimmu.2024.1384623)
Supplement: Supplementary file 1 [file DataSheet_1.docx]

Supplementary Material

Myelomodulatory treatments augment the therapeutic benefit of oncolytic viroimmunotherapy in murine models of malignant peripheral nerve sheath tumors

*** Correspondence:**

Siddhi N. Paudel, PhD

Center for Childhood Cancer Research

The Abigail Wexner Research Institute at Nationwide Children’s Hospital

700 Children’s Drive

Columbus, OH 43205, United States

Tel: 614-722-2200

[paudelsiddhi@gmail.com](mailto:paudelsiddhi@gmail.com)

# Supplementary Figures


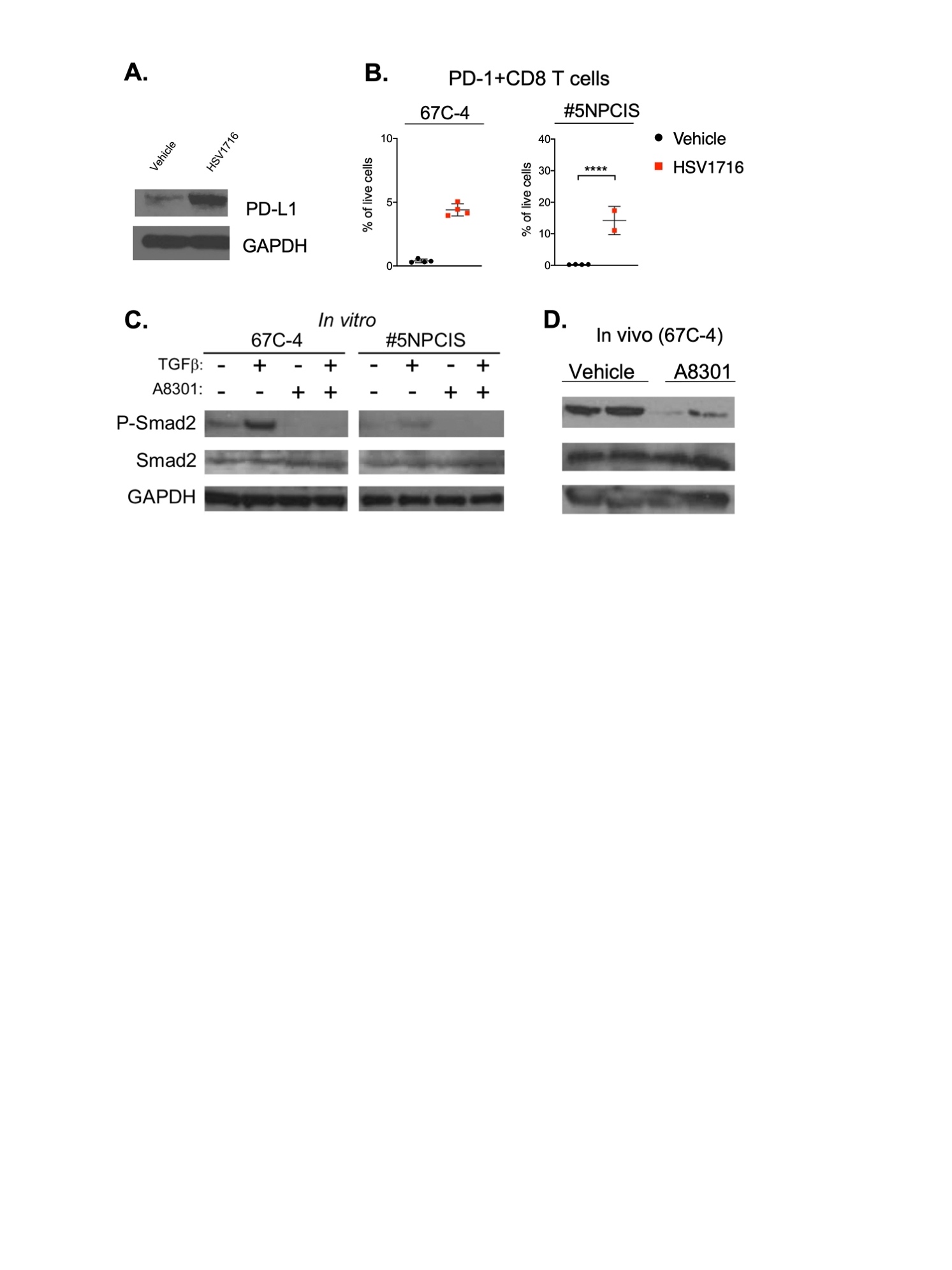


Supplementary Figure 1

MPNST mouse models express PD-1/PD-L1 and TGFβ similar to human patients.

Mice bearing #5NPCIS or 67C-4 tumor were given three intratumoral 1x10^8^ PFU doses of HSV1716 or an equivalent volume of PBS on day 0, 2 and 4. They were sacrificed at days 5 and 7 and stained for A) PD-L1 expression in #5NPCIS (immunoblot) and B) PD-1^+^CD8 T cells in 67C-4 and #5NPCIS (flow cytometry). C) MPNST cell lines were exposed to TGFβ in presence or absence of small molecule A8301 which blocks TGFβ receptor signaling and analyzed by immunoblot for phosphorylated Smad2. D) Mice-bearing 67C-4 tumors were treated with A8301 and analyzed by immunoblot for phosphorylated Smad2. Both cell lines and 67C-4 tumors were found to have intact TGFβ signaling that can be effectively inhibited by A8301.


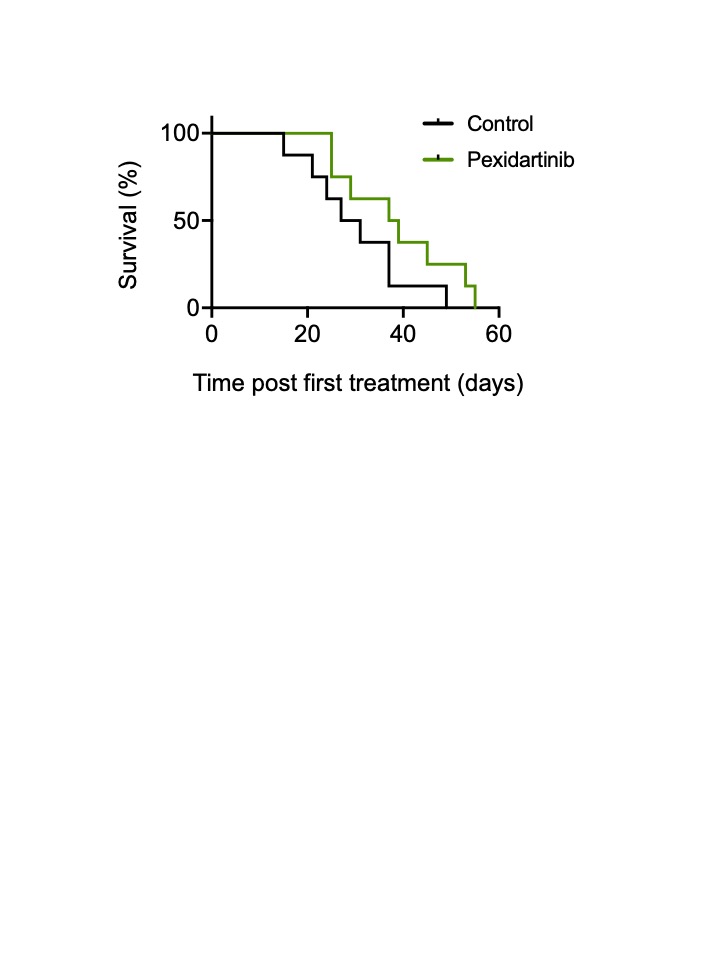


Supplementary Figure 2

Pexidartinib does not have any significant effect on 67C-4 murine model as a monotherapy.

67C-4 tumor-bearing mice were treated with 50 mg/kg of pexidartinib or vehicle control via oral gavage every alternate day until the experimental endpoint. The statistical significance of survival data was assessed using the log-rank test (n = 7 to 8 for each experimental group).
